# Youth perspectives on mental health during COVID-19 lockdown in a Small Island Developing State: implications for emergency response

**DOI:** 10.3389/fpsyt.2023.1285399

**Published:** 2024-01-05

**Authors:** Madhuvanti M. Murphy, Cecile Pemberton, Erica Wheeler, La Donna Gulston, Odessa Kerr-Layne, Ayana John, Beverly Baksh, Gail Thomas, Caroline F. Allen

**Affiliations:** ^1^The George Alleyne Chronic Disease Research Centre, The University of the West Indies, Bridgetown, Barbados; ^2^QURE Ltd., Arima, Trinidad and Tobago; ^3^The Pan American Health Organization/World Health Organization, Port of Spain, Trinidad and Tobago; ^4^The Ministry of Health, Port of Spain, Trinidad and Tobago

**Keywords:** youth, mental health, pandemic, lockdown, COVID-19

## Abstract

**Introduction:**

Evidence exploring the relationship between COVID-19 mitigation measures and mental health has primarily been from quantitative studies in large, developed countries. A qualitative study to explore the knowledge, attitudes and behaviors of young people living in Trinidad and Tobago was conducted to engage and collaborate with youth on matters affecting them during the pandemic.

**Methods:**

Ten virtual focus groups were conducted with 64 participants aged 18 to 24 in 2021 when partial lockdown measures were in effect for COVID-19 prevention. Groups were stratified by geographic location and socioeconomic status. The recordings were transcribed and analyzed to explore themes of importance to youth.

**Results:**

Negative impacts on mental health emerged as a strong theme. Lack of timelines for restrictions led to wide ranging mental health impacts, conflict and tension existed in home environments, longer restrictions led to erosion of the social culture, and young people experienced stress about the changing face of education and job security due to the pandemic.

**Discussion:**

Measures taken to address one serious public health concern, COVID-19, led to the aggravation of another serious public health concern, mental ill-health. Mental health initiatives to help young people navigate issues specific to their generation must be developed. In low resourced Small Island Developing States settings. The increased need for mental health services during and because of the COVID-19 pandemic highlights the need for strengthening the capacity and resilience of these to respond to environmental and health emergencies. Building the resilience of educational and employment services is also needed.

## Introduction

The COVID-19 pandemic has caused massive disruption to populations across the world. Health impacts for persons infected range from asymptomatic through respiratory and other symptoms to death, with the severity of illness varying according to factors including age and pre-existing health conditions.

In many countries non-pharmaceutical interventions (NPIs) were employed as preventive measures, including lockdowns, curfews, quarantines, “stay-at-home” measures, travel restrictions and border closures and involving temporary or partial closing of institutions such as workplaces, schools, leisure and sports facilities. NPIs are usually implemented when medical interventions are absent or severely inhibited (1). In the early pandemic period in 2020-21, when little was known about the virus and in the absence of effective vaccines, many countries relied on NPIs to prevent transmission.

The Caribbean comprises multiple islands and low-lying mainland territories and countries. The Region is remarkably diverse, with a mix of languages and ethnicities. Countries have varying sizes, geographic landscapes and political systems. Population sizes vary from extremely small (approximately 2,000 in Saba) to small (approximately 11.2 million in Cuba). Many of the countries are Small Island Developing States (SIDS), which have been identified as facing multiple vulnerabilities from natural and other disasters and are highly prone to external economic shocks more so than developed countries (2). SIDS such as those in the Caribbean are limited in their capacity to prepare for and respond to acute environmental and health emergencies such as the COVID-19 pandemic (3). Most SIDS have limited human resources for emergency response and have high import dependency for food and medical supplies (4). Caribbean SIDS had to compete with larger developed countries for pandemic-related supplies such as personal protective equipment (PPE), ventilators, testing kits, laboratory reagents and vaccines. In common with other SIDS, they struggled with supply chain issues. When vaccines became available, there was a high degree of hesitancy in taking them (5). For these reasons, the public health response throughout the region had a heavy focus on NPIs throughout the pandemic (6). Globally, lockdowns have been reported to have wide ranging undesired consequences and negative social impacts including unemployment, loss of livelihoods, educational disruption, gender-based violence and mental health crises (7–9). Similar effects were felt across the Caribbean region with increases in unemployment averaging 27%, and with estimates of over 300,000 job losses across the Eastern Caribbean area due to the pandemic, along with an estimated 6-fold increase in severe poverty (9).

The Republic of Trinidad and Tobago (T&T), a SIDS in the Caribbean region with a population of 1.4 million, reported its first case of COVID-19 on March 12th 2020. On June 1st 2021, approximately 15 months later and immediately before this study was conducted, there were a total of 24,314 confirmed cases of COVID-19 and 507 deaths from COVID-19 in T&T (10). The Government of T&T implemented various strategies aimed at limiting movement and by extension limiting COVID-19 transmission, ranging from social distancing measures within the population to closure of international borders on March 22nd 2020 (just 10 days after the first recorded case), a “stay at home” order (i.e., full lockdown) implemented on March 29th 2020. This was eventually eased to a state of emergency and night-time curfew (from 9 pm to 5 am) on May 16th 2021 (11).

Partial and full lockdowns are defined by the Assessment Capacities Project (ACAPS) Government Measures. Partial lockdown includes that (1) The population cannot leave their houses apart from for specific reasons that they have to communicate to the authorities and (2) All stores that are not related to alimentation or pharmacies are not open. Full lockdown includes that (1) The population cannot leave their houses apart for specific reasons that they have to communicate to the authorities and (2) All non-essential services are closed and production stops (12). In T&T, full lockdown was initially implemented for 6 weeks, and this was followed by partial lockdowns with varying levels of severity and stipulations. Sports events were banned and beaches closed to the public. Borders were closed, with gradual easing to permit the entry of T&T nationals with negative COVID-19 test results. The various lockdown measures had no clear end date and were modified periodically, for example with respect to the numbers of people allowed to gather. Unemployment rose among those not regarded as essential workers (such as emergency services, health care staff and supermarket staff) and those unable to work from home. In T&T pre-pandemic youth unemployment was almost triple the overall unemployment rate, with an estimated 27% increase in unemployment during the pandemic (9). Related to mental health, a cross sectional study conducted with 385 adults attending mental health services in (T&T) found that in response to the lockdown measures, approximately 42% of men compared to 22% of women found it difficult to stay at home, while 52% of women reported feeling stressed compared to 38% of men (13).

Available global evidence suggests that young people, defined by the World Health Organization as persons ages 10–24 (14), are at lower risk than older adults, of severe symptoms of COVID-19 (15). Despite this, for young adults in the US who tested positive for COVID-19 and had no chronic health issues, 20% had not returned to their usual state of health after 2–3 weeks (16). Data on the second wave of COVID-19 in Europe indicated that the highest incidence was in young adults aged 18–29 (17). Early reports from the UK during the COVID-19 pandemic showed that while 71% of the general population reported changing behavior in response to government guidance, behavior change was notably lower for young adults ages 18–24 years (53%). These findings indicate a need for targeted communication to this group (18). Research examining the effects of COVID-19 on youth has found that the loss of structured schedules and activities related to education and employment led to detrimental psychosocial effects (19–22). A study with 104 university medical students in the Caribbean found that 30.8% of students were extremely anxious during lockdown, while 26.9% reported being extremely depressed during the lockdown (23), similar to evidence from other parts of the world showing many indirect consequences of COVID-19 on young people due to anxiety and stress (19, 24, 25). Most of the existing evidence exploring the relationship between COVID-19 mitigation measures and mental health have been quantitative studies in larger developed countries that may have different cultural and social norms around mental health (26, 27).

There are very few studies of COVID-19 and mental health in the Caribbean, especially among young people. There is also a lack of qualitative research that explores young peoples’ experiences in SIDS during the pandemic and how they responded to NPIs and other aspects of the pandemic-related environment. Based on these gaps, a qualitative study was conducted to explore the COVID-19-related experiences, knowledge, attitudes and behaviors of young people in T&T to inform communication with youth and to strengthen services in response to their needs.

## Methods

Data was collected between June and August 2021 from young people aged 18 to 24 who had been living in either Trinidad or Tobago during the COVID-19 pandemic (i.e., since March 2020). Ten virtual focus groups were conducted on the Zoom online conferencing platform. Focus group discussions were chosen as the data collection method as they allow for discussion and interaction among participants, allowing a broader understanding of the issues affecting the population of interest than individual interviews. A major objective of the study was to develop communication strategies with youth, and focus groups are a commonly used method in communication development.

The focus group guide included questions on (i) knowledge about COVID-19 in general and regulations related to COVID-19 in T&T, (ii) attitudes towards COVID-19 in general and regulations related to COVID-19 in T&T, and (iii) behaviors before COVID-19 and during various stages of the COVID-19 pandemic in T&T. The World Health Organization’s Risk Communication and Community Engagement (RCCE) Action Plan Guidance, COVID-19 Preparedness and Response (28) was used to develop questions for the data collection instrument.

Focus group discussions lasted between 78 and 123 min. Anonymity was stressed to encourage participants’ candor and honesty about their beliefs and behavior by minimizing fear of legal or other repercussions. To protect identities, participants were asked to use pseudonyms throughout the focus groups, and no video feed or display pictures were used.

Recruitment was conducted through gatekeepers at 15 local organizations who had access to and were trusted by young people in various communities. Digital flyers and electronic screening forms were widely distributed through these organizations. Participants were recruited purposefully based on geographic region [North West Trinidad (NW), North East Trinidad (NE), Central Trinidad (C), South Trinidad (S), and Tobago (T)] as well as socioeconomic status (SES). An SES index was constructed. To assign participants into a lower or higher SES group for the purpose of this study, the researchers settled on an SES index based on two constructs – education and assets (as a replacement for income) (29, 30). The SES index had scores ranging from 0 to 11 based on level of education plus access to specified assets in the household of participants (e.g., a clothes dryer, vehicle, broadband Internet). Participants who had scores of 6 and lower on the index were placed in Group 1, while participants with scores of above 6 on the index were placed in Group 2. Geographical region and SES were chosen as the stratifying variables in this study to enhance the geographic and socio-economic coverage of the study, while enabling tentative comparisons between groups.

Two focus groups were conducted in each region, and participants were assigned to each group based on SES which was determined during screening. Other basic demographic data was also collected from participants during screening to aid analysis. Participants were provided with mobile phone credit as compensation for participation. The focus group recordings were transcribed verbatim, and the recordings destroyed thereafter to preserve confidentiality. Data were analyzed by using a thematic framework. A coding scheme was developed by researcher MMM with both inductive (based on the focus group guide questions) and deductive (based on emerging themes) codes. For example, codes related to the focus group guide included “knowledge about COVID-19; coping mechanisms; perception of lockdown regulations; positive behaviors in response to lockdown; negative behaviors in response to lockdown.” Examples of deductive codes included “consequences of the pandemic; job loss; perceived mental health; family dynamics.” The transcripts were managed using Atlas.ti qualitative software, and coded by MMM and then discussed with CFA to ensure accuracy. Coded transcripts were compared and contrasted for emerging themes which were discussed with the entire research team to reach consensus. Data saturation was achieved, that is, findings across focus groups were becoming repetitive as we approached our final focus groups.

Following completion of the study report, the organizations involved in initial recruitment were contacted to invite young people to an online meeting to discuss the findings and initial recommendations. The eleven youth participants in the meeting provided feedback which has been reflected in the discussion.

## Results

The 10 focus groups comprised of a total of 64 participants. Two thirds of participants were female although males were represented in all groups, and 45% of participants identified as African descent. All participants had completed at least secondary school, with half having completed University. While 46% of participants were students, another 31% were unemployed. Approximately 75% lived in households with other people and of those more than half lived with parents. The groups were equally divided with regard to SES (see Table 1).

**Table 1:** Participant demographics.

Gender	Ethnicity	Education	Employment	Living arrangements	SES
21 Male	38 African	32 Secondary	14 Employed	2 Alone	33 Low SES (1)
43 Female	8 East Indian	32 University	20 Unemployed	47 With Parents	31 High SES (2)
	18 Mixed		30 Student	15 Other	

There were some common findings across locations, gender and ethnic groups. While in some locations, responses differed between the two socio-economic groups, the associations with either “high” or “low” SES were not consistent across locations. There were no clear differences between locations in the issues raised and the responses and recommendations of participants. Youth were generally very knowledgeable about COVID-19 transmission and prevention, and for the most part followed the government guidelines, even though a few participants did not agree with them. What deductively emerged from the discussions was the perceived impact of one specific NPI, lockdowns, on mental health.

### Behaviors in response to the COVID-19 lockdown restrictions had more negative than positive mental health implications

#### Lack of timelines for restrictions led to wide-ranging mental health impacts

Although most participants stated that they had been adhering to the lockdown restrictions set by the government, mainly because of fear of legal repercussions, they found that not being able to leave their homes as well as not knowing how long the restrictions would be in place for were causes of anxiety.

*“It's like a year and six months now that we have been more or less locked up inside. Not being able to function normally as a country”* (C2, Female).

The seemingly never-ending timeline of the pandemic led to feelings of loneliness, depression and fear:

*“I believe a lot of persons are scared, I believe a lot of persons are just wondering, when will it end? Because you know, we have been hearing, ‘Okay – this is the new normal, but we want to get things back to how it was before.’ So you know, you have that hope, you're looking forward to getting things back to how it was before. But of course with the new variants and with COVID-19 evolving, it's just as though… okay… would this thing ever end?”* (T1, Female)

#### Home environments were not always a sanctuary

As most participants lived with family members during the pandemic, they spoke about navigating spending time with others in the household and alone time. Interestingly, the additional time spent with family members being at home was not always seen as a positive experience. The issues of being home spanned a wide range. Some people spoke of the inconvenience of being cooped up with family members they did not get along with:

*“Not everyone's family gets along, yuh know, and has the best family – sit down and play board games together and stuff [laughs] so, yuh know, there's a lot more like… tension and everybody just kind of, you know, stays in their own room, and in their own space. So, you know, it's not… it's kind of uncomfortable to be home every day. You know, with people who doh really get along and stuff like that.”* (NE2, Female)

Others spoke about more serious matters such as abuse occurring in household. Some young people were now forced to stay in toxic environments with no outlet and limited outside support. Participants voiced concern that these issues were not being openly discussed and therefore there was little that young people in these situations could do:

*“I know a lot, a lot of young people actually not coping well, because a lot of them, the household, the domestic violence, the abuse and it doesn't even have to be physical abuse, it could be emotional abuse, you know? Some youth really, actually, can't handle it. They need to be outdoors, or they need to be with friends and due to the COVID, they can't do that. A lot of people, they're not speaking about that. The abuse, emotional abuse, physical abuse, the mental abuse, a lot of people not speaking on that… I've seen it happen, that they [young people] can't cope with that life… As a youth if I was going through something like that, and this is so personal, but the suicidal thoughts might come to mind or you know, like, the drug abuse might come to mind”* (C1, Female)

Thus, the mental health impacts being felt ranged from stress or frustration all the way to thoughts of suicide or drug use as coping mechanisms for dealing with consequences of the restrictions.

#### Longer restrictions led to erosion of the social culture

Young people from all groups spoke about culture and how it was affected by lockdown experiences. They discussed the loss of time spent with friends and family, being out ‘liming’ (a local term for ‘hanging out’). Not being able to have these face-to-face social interactions which were a common part of everyday life led to feelings of depression, anxiety and frustration:

*“I agree [with the other participant] when she says it restricted our lives, but also it has led to a high sense of depression in most of my young friends I realize. Also, lack of contact with persons… we missing [our] friends. Whole time we on lockdown we can’t go nowhere and we feeling frustrated.”* (S1, Male)

Participants reported that there was an increase in the use of digital technologies among the young to compensate for the lack of physical interaction. Social media platforms such as Discord, WhatsApp, Instagram, TikTok, Twitter and Facebook were used to stay in contact with friends and family. This included having online parties:

*“I utilize this online platform to have interactions like play games with my friends…and sometimes we randomly call each other as a family and just talk and laugh using online platforms”* (S2, Male)

As some of the restrictions related to lockdown were relaxed to allow small groups of people to interact, youth began socializing again, which gave some relief, even though they missed being out at social activities in big groups such as at parties or large outdoor sporting and other activities:

*“I see some girls doing game nights. For some reason, game nights are a trend these days. And plenty people, instead of going somewhere to lime, they would arrange something and go to a guesthouse for a weekend or a day or something. They would have a… I wouldn't say an event… just a day where they lime and rock back or whatever.”* (NW1, Male)

But then full lockdown restrictions, which included not going to the beach, were reimplemented due to surge in COVID-19 cases. The fluctuations in restrictions were seen as a mental rollercoaster. One participant, although she felt that others would judge her, shared her own experience with disobeying the regulations, justifying her decision based on having followed the rules for so long, and needing the mental and social escape. In order not to disregard the restrictions completely, she limited the number of people she interacted with to five:

*“Me and my work colleagues, just like five of us, did have like a lil something. Some house pool like something… That's this year, actually, because you know, last year, alright, I followed the regulations, I said, "Yeah, alright, we will get to [do something]… The country will open back up, I will get to go to the beach or something for my birthday." And then boom we just locked right back down for my birthday. I cried, you know, I was really depressed because I just wanted to do something, go somewhere, dip in a pool or beach or something and I love the water. That was really depressing for me personally, so I had to do something and I did… I rented a guest house, and we spent one night, and that was it. Well, 24 hours basically. We cooked, we drank, we played cards. We talk [nonsense] and we had fun. And that was for me.’* (NW2, Female)

As time went on, the level of frustration with the lockdowns increased and compliance eroded. Particularly around the Easter holiday period in early April 2021, more young people were perceived to have had enough and were openly flouting the restrictions:

*“Well, not everybody, but a lot of people, a lot of young people, but you see after a point in time persons kind of got fed up, especially around Easter this year. And we saw after Easter this year there was a huge surge in the virus, right? So I believe, you know, persons just kind of got fed up. Is like, okay, doing this thing for so long, you know, let me just free up for once, so I believe now and from since Easter to now, you know, persons have just been, I guess, living their life.”* (T1, Female)

#### Lockdown allowed for self-improvement and self-care

Young people reported some positive mental health impacts. Lockdown was seen by some participants, both male and female, as an opportunity for self-reflection and empowerment. A few participants spoke about using the time to learn new skills, take courses, or start entrepreneurial ventures. Others saw it as a time to engage in relaxing activities such as planting and gardening, and seeking spirituality, which they would not have had time for pre-pandemic.

*“It actually helped me, on a personal level, to enhance my spiritual life, to improve my spirituality. Like I taking more time to see about myself now and actually disconnecting from social media more and taking more time to take care of myself. Instead of using the Internet to do ah set of nonsense, I using the Internet to research things that will help to benefit me and uplift me and to help take me through a time like this, because things rough right now. So, obviously if you want to survive, yuh hadda start taking measures and putting things in place to help yuh survive.”* (T2, Male)

Physical activity was discussed in nearly all groups, with some participants seeing the time at home as a reason to engage in more physical activity, while others spoke about not wanting to engage in any kind of physical activity, even if they had been active before the lockdown. These were all seen as coping strategies to deal with being at home for extended periods. Unfortunately, positive feelings generally waned as time went on and there was no indication of when movement restrictions would end.

### Young people were particularly stressed about the changing face of education and job security due to the pandemic

#### Online fatigue affected education

With 78% of participants being either unemployed or in school, finding job opportunities post-pandemic was first and foremost on many of their minds. Nearly half of the youth interviewed were enrolled in tertiary education and while they saw the quick transition to online classes as a positive change, many struggled with online learning formats and found being online frustrating, particularly for programs that had practical components. This was in part because they spent most of their free time online as well, whether gaming or on social media, leading to online fatigue.

*“We had to learn online and that took away aspects of it, which was very frustrating. Because how yuh gonna learn something that's supposed to be practical through a screen? My degree didn't fully move online like a lot of people – it still had its limitations. Yeah, for months last year we were online, and is just frustrating.*”(NE2, female)

#### Stressful feelings related to lack of practical experience affecting job prospects

The main concern for those in school was the ability to secure internship opportunities or jobs upon completion of their degrees, particularly since many people were now working from home or had lost jobs. This was a source of stress as they wanted to be independent of parents and other family members. They felt that being at home deprived them of work experience and communication skills, and this could reduce their employment prospects.

*“It's basically trying to find a job, getting a job. I just finish secondary school, so you're out there and you have to get money somehow. You can't expect to be dependent on your parents all the time to be doing something for you. Finding a job for me will be kind of hard because most people want people with experience. And then, I was home, basically for the entire year, I did not go out, only for CXC. And then, I don't know how to communicate properly with people in real life, I can only communicate with people now online properly.”* (NW1, Female)

#### Unemployment within the household also led to feelings of stress

Some youth described feeling stressed about their household financial situation when they were unable to contribute:

*“It affects me, reason being, I'm not able to work. And because I live with my mom, it kinda hard on her because I have other siblings. If it is I was working, I'd be able to contribute and bring stuff to the table, rather than just, being there and depending on her for everything, which is hard.”* (T2, Female)

Equally stressful were situations in which other family members had been laid off or had their work reduced during the pandemic. This reduced the income available at household level and increased stress within the household.

### The youth struggled with finding coping and support mechanisms

Participants struggled to understand how to cope with the emotions they were feeling during lockdown.

*“I think that most young people are not sure how to deal with it. So, they just probably stay in bed whole day and be depressed rather than trying to deal with their emotions.”* (S1, Female)

One youth who had been offering mental health support online during the pandemic, noted that mental health was not adequately addressed through social support or service provision prior to the pandemic. He argued that because of this, some end up using unhealthy coping strategies such as drug and alcohol use.

*“I definitely think that mental health has been a fad before, and I use the word ‘fad’ specifically because people don't champion it in Trinidad as a serious thing. It's more like ‘oh suicide prevention’, whatever, “like” a post or whatever. So, I think people are cognizant of the fact that yeah, you need to take care of your mental health, but at the same time, they've not been given the correct tools to take care of their mental health and therefore as much as they get depressed, they probably don't know how to deal with it. So, I would imagine that drinking would have risen, I saw that smoking weed, from things that I've read, and what I've heard, etc. would have gone up as well to cope with the depression.”* (C2, Male)

The same participant noted that he was involved in providing a level of mental health support but that the service provision landscape was inadequate and access was limited.

*“I do a lot of mental health stuff on Twitter, whatever. I share as much information as I can. I had community service, digital community service groups like four times a week to at least help people. So there have been services – it's just like – you have to find. Because I'm sure I'm not the only person with those kind of services out there. It's just the question really boils down to… if you are dealing with like loneliness or depression… number one, are you seeking out healthier methods? Number two, do you have access to those things? So, to say I know to what degree people have been coping well, I don't know. I could only say like on my end at least, at my point on the internet, I've been trying to help fight that.”* (C2, Male)

In a similar vein to what this young man said, a participant in another focus group noted that despite ‘the cry for help’ from young people, mental health had not been prioritized during the lockdowns. Recommendations from the youth participants themselves focused on the need for mental health initiatives specifically for young people to help navigate the issues specific to their generation:

*“You're hearing a lot of people, young people are complaining about, like, a lot of mental issues, social issues, whether it's abuse, or just depression, anxiety. But I don't think I have seen a big mental health initiative and push during this pandemic, especially for young people. And I think that's kind of a concern. Because an issue that you're seeing is everyone's focusing on COVID COVID COVID and a lot of people are just struggling… the side effects of COVID. So I think that's a big thing to probably recommend.”* (NE2, Female)

## Discussion

Previous research regarding NPIs found that lockdowns implemented in Caribbean countries during the initial outbreak of COVID-19 (March through May 2020) led to large reductions in community mobility; more than 60% mobility reduction in most countries. This was higher than in most countries around the world used for comparison (31). This qualitative study found that the severe lockdowns imposed in Trinidad and Tobago were perceived by participants to have had a positive impact on curbing the spread of a viral disease but to have had severe negative impact on mental health. Therefore, measures taken to address one serious public health concern led to the aggravation of another serious public health concern. The implications of the study findings with accompanying recommendations are further discussed below.

NPIs are an inexpensive and effective option of curbing the spread of disease during an epidemic or pandemic, or preventing injury during natural disasters, in resource-constrained SIDS. A specific set of NPIs, lockdowns, particularly when implemented with indefinite time periods and unpredictable stipulations, interfere with social support mechanisms and bring mental health challenges which require management (32). Understanding the impact on youth mental health of NPIs which may be implemented as a first course of action during emergency situations, can assist in developing appropriate programming and communication for youth to reduce the negative impacts on mental health, given that youth mental health problems can set the tone for more serious mental issues moving into adulthood. It is therefore imperative that these issues emerging from the COVID-19 pandemic, and particularly the lockdown, are considered and addressed for future emergency responses (19).

Youth should be involved in development and design of mental health services that are appropriate to their age and context (33). This may involve a combination of in-person, telephone hotline and online services. Some participants suggested that the creativity of youth, including artistic and entrepreneurial skills, can be used in the design of messaging and communication and in seeking opportunities for young people in the midst of challenges. Their networking and social support skills should be nurtured in developing a variety of services. Civil society initiatives can usefully complement public services.

The increased need for mental health services during and because of the COVID-19 pandemic may be difficult to address in SIDS setting which are low resourced unless more training and capacity is provided for mental health care, particularly as similar Caribbean countries have reported that mental health services have already been overwhelmed (34). Even given SIDS’ resource constraints, the government should prioritize the necessary support for youth initiatives in terms of skills building, technical and targeted financial support. Further developing directories of services, such as the existing FindCareTT directory of mental health and psychosocial support services developed by an alliance of governmental and non-governmental agencies, can help enhance access and establish the necessary referral networks. In a region also prone to tropical storms and earthquakes the solutions should not depend solely on Internet availability. For instance, there has been some success in addressing limited mental health capacity in SIDS such as psychological first aid courses for health care workers and the general population done in response to the risk of disasters such as severe tropical storms and hurricanes (35, 36).

Also affecting youth mental health was the impact on learning and the procurement of jobs and income as a means of gaining independence and transitioning into adult life. Our qualitative work corroborates survey research conducted early on in the pandemic by the International Labor Organization (37), which highlighted challenges such as the absence of social contact and group work learning having switched to remote learning and the reduced effectiveness of the school-to-work transition for young people due to the pandemic. It concluded that the negative mental health symptoms described by young people, who are still in a period of development, may delay employment outcomes (38). These issues clearly continued, and possibly worsened with the extended lockdowns in T&T. Recommendations to address these issues include improved job counseling for youth and work with employers to provide additional job experience, internships and mentoring to take into account how education and skills have been affected by the pandemic (37, 39). The design of virtual education needs continued attention to engage young people and combat the online fatigue described by many youth (40, 41).

It is important to frame findings in the context of the participant population. Regardless of SES, participants were well educated, and also had internet access in order to participate. Therefore it can be assumed that they would have more access to information and resources than those who may not be as educated or have access to communication channels afforded by the Internet. Furthermore, it is likely that those without access to resources, particularly those in vulnerable communities, may have been more affected in terms of reduction of social networks and communication channels, leading to even more negative mental health impacts. Also, while youth described feelings of anxiety, depression and other symptoms of mental health, they were not asked to disclose if they had been medically diagnosed for any mental health symptoms illness, although this does not diminish the experiences and feelings described.

Potential limitations to the study include the listing of the Ministry of Health of Trinidad and Tobago, which gave ethical approval for the study, on recruitment materials. Some people may not have participated for fear of negative consequences by the government if they disclosed certain behaviors in the context of public health restrictions. For those who did participate there was the possibility of bias in the responses given as persons may not admit to breaking the movement restrictions for fear of legal repercussions. We do believe this limitation was alleviated though, as measures were taken to protect identities so that everyone could speak freely and there was no indication during analysis of any inconsistencies in what participants reported. The use of an online platform to conduct the focus groups may have also affected who participated. One gatekeeper described youth as “Zoomed out” given that school and employers were routinely using online platforms during the pandemic routinely. Further research is needed to delve deeper to understand the issues being faced by vulnerable groups in Trinidad and Tobago to ensure services provided for mental health can meet their needs.

This study was the first to explore knowledge, attitudes and behaviors of young people during the COVID-19 pandemic using qualitative methods in Trinidad and Tobago or the wider Caribbean region, and which elicited youth concerns around mental health. Given the vulnerability of SIDS to public health and other emergencies and disasters and to address ongoing mental health challenges among youth, it is critical to strengthen mental health services responsive to their needs. More specifically, interventions need to alleviate the dire mental health consequences arising from emergencies that could impact youth as they transition to adulthood. Building the resilience of educational and employment services is imperative to successful interventions.

## Data availability statement

The raw data supporting the conclusions of this article will be made available by the authors, without undue reservation.

## Ethics statement

The studies involving humans were approved by (1) the Ministry of Health of Trinidad and Tobago and (2) the PAHO Ethics Review Committee. The studies were conducted in accordance with the local legislation and institutional requirements. The ethics committee/ institutional review board waived the requirement of written informed consent for participation from the participants, who were all adults, because data collection was conducted online due to COVID-19 restrictions. Participants were informed of the general guidelines and expectations for focus group participation when they were called to schedule their session. In addition, a digital copy of the informed consent form was sent (via WhatsApp) to each participant in advance of the focus group session. At the start of each focus group, the Informed Consent Form was also read aloud to the participants who had decided to attend. Participants were invited to have any queries settled at that point by the trained facilitator. Before the focus group session started, verbal consent was sought individually from each participant. Their verbal confirmation of consent was audio-recorded.

## Author contributions

MM: Conceptualization, Formal analysis, Methodology, Writing – original draft, Writing – review & editing. CP: Formal analysis, Investigation, Writing – review & editing. EW: Conceptualization, Funding acquisition, Writing – review & editing. LG: Conceptualization, Writing – review & editing. OK-L: Conceptualization, Writing – review & editing. AJ: Conceptualization, Writing – review & editing. BB: Conceptualization, Writing – review & editing. GT: Conceptualization, Writing – review & editing. CA: Conceptualization, Formal analysis, Supervision, Writing – original draft, Writing – review & editing.
